# Bias and discriminability during emotional signal detection in melancholic depression

**DOI:** 10.1186/1471-244X-14-122

**Published:** 2014-04-27

**Authors:** Matthew Hyett, Gordon Parker, Michael Breakspear

**Affiliations:** 1 Systems Neuroscience Group, QIMR Berghofer Medical Research Institute, 300 Herston Road, Herston, QLD 4006, Australia; 2 School of Psychiatry, University of New South Wales, Prince of Wales Hospital, Hospital Road, Randwick, NSW 2031, Australia; 3 Black Dog Institute, Prince of Wales Hospital, Hospital Road, Randwick, NSW 2031, Australia; 4 The Royal Brisbane and Women’s Hospital, Herston, QLD 4029, Australia

**Keywords:** Bayesian analysis, Decision-making, Depression, Melancholia, Signal detection

## Abstract

**Background:**

Cognitive disturbances in depression are pernicious and so contribute strongly to the burden of the disorder. Cognitive function has been traditionally studied by challenging subjects with modality-specific psychometric tasks and analysing performance using standard analysis of variance. Whilst informative, such an approach may miss deeper perceptual and inferential mechanisms that potentially unify apparently divergent emotional and cognitive deficits. Here, we sought to elucidate basic psychophysical processes underlying the detection of emotionally salient signals across individuals with melancholic and non-melancholic depression.

**Methods:**

Sixty participants completed an Affective Go/No-Go (AGN) task across negative, positive and neutral target stimuli blocks. We employed hierarchical Bayesian signal detection theory (SDT) to model psychometric performance across three equal groups of those with melancholic depression, those with a non-melancholic depression and healthy controls. This approach estimated likely response profiles (bias) and perceptual sensitivity (discriminability). Differences in the *means* of these measures speak to differences in the emotional signal detection between individuals across the groups, while differences in the *variance* reflect the heterogeneity of the groups themselves.

**Results:**

Melancholic participants showed significantly decreased sensitivity to positive emotional stimuli compared to those in the non-melancholic group, and also had a significantly lower discriminability than healthy controls during the detection of neutral signals. The melancholic group also showed significantly higher variability in bias to both positive and negative emotionally salient material.

**Conclusions:**

Disturbances of emotional signal detection in melancholic depression appear dependent on emotional context, being biased during the detection of positive stimuli, consistent with a noisier representation of neutral stimuli. The greater heterogeneity of the bias across the melancholic group is consistent with a more labile disorder (i.e. variable across the day). Future work will aim to understand how these findings reflect specific individual differences (e.g. prior cognitive biases) and clarify whether such biases change dynamically during cognitive tasks as internal models of the sensorium are refined and updated in response to experience.

## Background

Melancholia is frequently conceptualised as a biological disorder encompassing disturbances of mood, motor function, thinking, cognition and perception [1,2]. Whilst cognitive impairments in melancholia have been investigated in detail [3,4], definitive identification of selective neurocognitive impairments has not been achieved. Given the pressing need to examine underlying perceptual and inferential processes in heterogeneous illnesses such as depression [5], it is increasingly recognised that a range of methodological approaches should be utilised in the analysis of neurocognitive data to more accurately capture the nature of disturbances across differing depressive syndromes. Such refined approaches have direct utility in enhancing understanding of group-specific psychophysical processes across sub-types of depression.

There is typically great inter-subject variability on tests of neuropsychological function in the major psychiatric illnesses [6]. Meaningful interpretations of brain function in specific disorders is difficult given such variability. This is further compounded by summarising an individual’s position on a performance continuum (as with summing errors on a task) in order to infer the presence or absence of cognitive impairments. Furthermore, commonly utilised neuropsychological tests in those with depressive disorders typically rest upon broad construct-level approaches (e.g. tests of ‘executive function’ or ‘attentional control’) that do not facilitate the development of theories regarding specific psychophysical disturbances in individuals. Despite such drawbacks in assessing cognition in psychiatric illnesses, significant advances have been made over the past 20 years in explaining human perceptual inference and action [7-9] using probabilistic statistical principles such as those developed through a Bayesian-based approach [10]. The Bayesian statistical modelling approach has been applied to individual and group cognitive data across multiple cognitive domains, including signal detection that is viewed as encompassing the processes of attention, decision-making and executive functioning [11,12]. Formally, the signal detection capacity of an individual can be influenced by prior beliefs (or internal models of the world) and the incoming sensory stream, generating that individual’s response profiles. This, in turn, provides an ideal platform through which to measure perception and inference. In the analysis of cognitive data, signal detection theory or SDT [13] allows modelling of the optimal detection of stimuli, through estimating discriminability and bias [14]. SDT gives rise to measures of discriminability – how easily signal (response) and noise (non-response) trials are distinguished – and bias, reflecting how well the decision-making criterion relates to the optimal criterion. Both constructs reflect an individual’s internal model of the sensorium and their prior contextual beliefs. Signal and noise trials of a task can be represented along a perceptual strength construct in SDT, referring to the strength of inference made to a particular stimulus – that is, the probability that a conclusion (decision/action) is true given its premises. Inferences during streams of trials are continuously monitored through sensory experience and evaluation, and may then be used to update decision criteria for subsequent task performance. Rouder and Lu [15] suggest it is reasonable to expect that on such tasks there will be significant participant-level variability in signal detection sensitivity, creating a need for statistical models that capture individual processes.

Inter-subject variability is rarely modelled in neuropsychological studies of depressed individuals. Moreover, commonly used aggregation methods have the potential to lead to statistical effect estimates that may poorly represent group heterogeneity [15]. Bayesian statistics offer the ability to pre-specify prior knowledge through the specification of priors [16]. A Bayesian approach to data analysis is also appealing in the setting of decision- making in the face of uncertainty because it embodies the same type of assumptions – and hence represents the same constructs – as emerging models of human decision-making [17,18]. When considering group data using SDT, individual subject variability can be modelled using hierarchical Bayesian techniques [15], allowing estimation of data-driven posteriors of mean bias and discriminability as well as their variance or precision (the inverse of variance) [11,12]. When cognition is variably disrupted, as arguably is the case in depression, inter-subject estimates of bias and optimal judgement may be influenced, which can ideally be modelled through hierarchical Bayesian SDT analyses. There are several reasons as to why such an approach may offer significant benefit.

In health, cognitive ‘priors’ can be viewed as personal beliefs that are optimised towards the most likely value of a given percept [19]. In depression, however, such processes may be suboptimal in different ways across individuals, extending variously across perceptual, inferential and performance domains. It has been suggested that depression is associated with distorted inference (e.g. “arbitrary inference”) at certain levels of severity (e.g. psychotic depression [20]), yet despite recent theoretical research with Bayesian modelling in depression [5,21] no studies have employed Bayesian statistics to model cognitive capabilities in depressive illnesses such as melancholia. Most studies to date have attempted to delineate underlying mechanisms of negative cognitive biases [22] based on the notion that depressed individuals have a characteristically negative view of the self, world and future [20,23]. Several studies have shown that attention is selectively drawn to negative information (e.g. [24]), and that memory of negative information is enhanced [25]. However, few studies have provided a formal quantitative framework for modelling individual level disturbances from empirical psychophysical data. While some studies (e.g. [26]) have established evidence for neurobiological correlates of response bias, it remains to be seen whether cognitive biases extend across depression as a whole or whether they are specific to given individuals or sub-set diagnostic groups. From the findings to date it is evident that there is an unmet need in elucidating basic mechanisms of neurocognitive dysfunction across individuals with depression.

We propose that biases in emotional stimulus processing in depression can be accurately captured through investigation of different depressive sub-types using a hierarchical Bayesian emotional SDT framework. Employing an emotional word ‘go/no-go’ task, which requires responding and inhibition of responding to serially presented, randomly sequenced positive, negative and neutral words, we hypothesised that each depressed sub-set would show less optimal responding (poorer discriminability) across emotional signal conditions as compared to the control group, but that the melancholic sub-set would show more difficulty in detecting true signal trials from noise trials, particularly on emotional signal conditions (i.e. lower sensitivity) compared with non-melancholic and control participants.

## Methods

### Sample

Participants consisted of 20 melancholic and 20 non-melancholic depressed individuals, recruited through a specialist depression clinic at the Black Dog Institute in Sydney, Australia. A healthy control group of 20 participants was recruited through the community. The study was approved by the University of New South Wales Human Research Ethics Committee and all study participants gave informed consent prior to taking part.

### Psychiatric and neurological screening

Exclusion criteria for healthy controls included a lifetime history of a mood and/or psychotic disorder as screened by the MINI [27]. Depressed participants were considered eligible if they had a current major depressive episode, but no (hypo) mania or psychosis identified on the MINI. Those with depression were additionally required to meet a current (past 7 days) level of depression severity of 11 or more on the QIDS-SR^16^[28], indicative of at least moderate depression severity. All participants were required to be fluent in English, and the age range for inclusion was between 18 and 75 years. Exclusion criteria for all participants consisted of current or past drug or alcohol dependence, current or past history of neurological disorder (i.e. neurodegenerative conditions, stroke, central nervous system infection, tremor), a history of brain injury with significant impairment (i.e. neurotrauma from haemorrhage, oedema, hypoxia), invasive neurosurgery and/or an estimated full scale IQ (WAIS-III) [29] score of below 80 as assessed by the WTAR [30]. An additional exclusion criterion for depressed participants was having received electroconvulsive therapy within the preceding six months. Current medication was recorded. In addition to the above screening, all participants completed the Global Assessment of Functioning (GAF) [31] and the State-Trait Anxiety Inventory (STAI) [32]. Patient groups were assessed for observable psychomotor disturbance using the CORE measure [33]. Screening was conducted by trained research assistants.

Delineation of depressive sub-types (melancholic versus non-melancholic depression) proceeded according to the clinical criteria presented by Parker et al. [34]. These criteria are based upon characteristic clinician rated scores (by trained psychiatrists in the current study) across a number of domains and include presenting clinical features (e.g. symptoms as well as signs of psychomotor disturbance), previous response to drug and non-drug treatments, developmental factors, personality factors and family history [1,2]. The focus of the current study on perceptual accuracy but not reaction time tempered circularity between diagnostic assignment (e.g. cognitive slowing in melancholia) and signal detection performance. Previous research [35] has shown the prototypic diagnostic approach used in the current study (involving symptom and non-symptom data) to be more strongly differentiating of melancholic and non-melancholic depression than use of the DSM-derived [31] melancholic specifier criteria which consider symptoms only.

### Neuropsychological testing procedures

All study participants took part in a brief neuropsychological assessment administered by a trained research assistant, with tests taken from the CANTAB [36], that included the Stockings of Cambridge (SOC), Intra/Extra-Dimensional Shift (IED), Rapid Visual Information Processing (RVP) and Affective Go/No-Go Task (AGN). All testing took place in a sound-attenuated room that housed a desktop computer running CANTAB eclipse version 3.0 software. Additional computer hardware (touch screen and response/press pad) allowed recording of behavioural responses to the stimuli. The focus on the present report is on the AGN.

**Figure 1:**
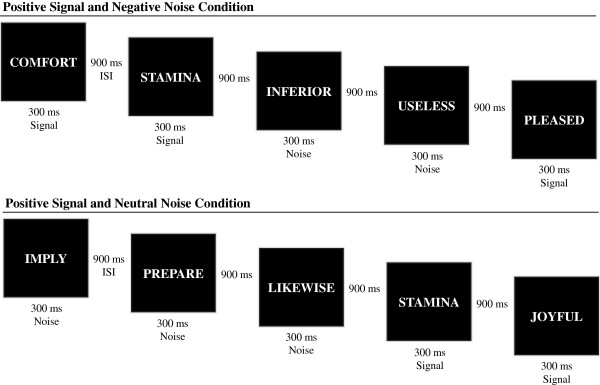
**Overview of task design showing positive signals with negative noise trials and positive signals with neutral noise – comprising the positive signal condition.** The same design – with varying noise – was consistent in the negative and neutral conditions.

The AGN is a test of emotional word discrimination that requires responses to ‘go’ trials, and inhibition of responses on ‘no-go’ trials, to negative, positive and neutral stimuli. The task consists of 20 blocks with 18 words in each block. The first two blocks are used for training and are not further analysed. Two word categories are presented within each block. No two consecutive blocks present the same word combinations. For each block there are nine signal trials and nine noise trials. Prior to the onset of each block participants are primed with what word categories to expect. As depicted in Figure 1, each block requires detection of signal and noise (positive, negative or neutral emotional word) categories, with six possible signal/noise combinations, in the following order of administration (repeated three times, giving the 18 blocks): positive (signal)-neutral (noise); positive–negative; neutral-positive; neutral-negative; negative-neutral; negative–positive.

Words appear for 300 milliseconds one at a time in the centre of the computer screen, followed by a 900 millisecond inter-stimulus interval (ISI). Each block hence lasts for 22 seconds. Participants rest between blocks for five seconds, allowing preparation for the following block. Analysis variables from the AGN consisted of hits (correct responses to signal trials), false alarms (incorrect responses to noise trials), misses (incorrect rejections to signal trials) and correct rejections (to noise trials).

### Hierarchical Bayesian modelling of AGN data

A hierarchical Bayesian graphical model for SDT (as detailed in [11,12]) was used for the AGN data. As a statistical technique, such modelling – with the use of Markov chain Monte Carlo (MCMC) sampling – allows for integration of a prior distribution (prior beliefs/knowledge) with data from the behavioural task to obtain approximations of the posterior distributions of the outcome parameters. AGN blocks were categorised by emotional signal condition (positive, negative, neutral): thus, three specified models were used for the current analysis. Alternating noise conditions were pooled together for each of the signal conditions. For example, a positive signal distribution in the current study had a noise distribution that included both negative and neutral stimuli. We modelled individual participant responses to the task (counts of hits and false alarms) to generate posterior estimates of discriminability and bias from hit and false alarm rates, separately for each of the emotional signal conditions, with uniform prior distributions hence assigning equal probabilities to all possible states.

**Figure 2:**
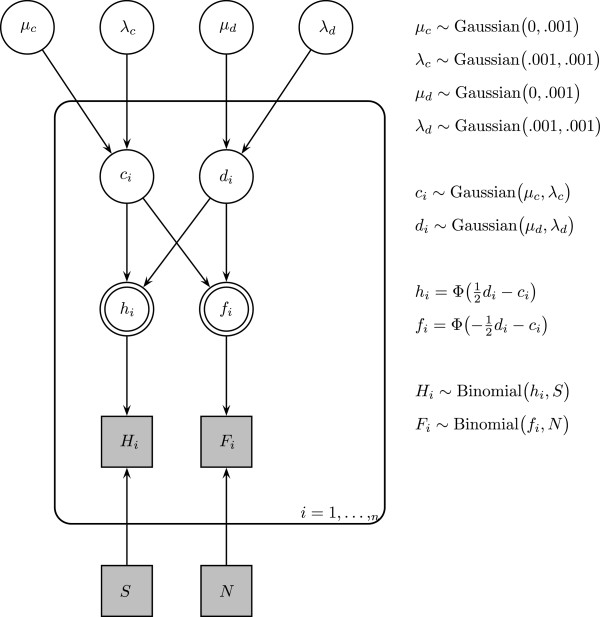
Graphical model for hierarchical signal detection theory.

The modelling approach is represented in Figure 2. By convention, unobserved variables are nodes without shading while observed variables are shaded, with continuous variables represented as circular nodes and discrete variables as square nodes. It thus follows for the current model that the observed behavioural data are represented by the shaded grey squares, and our estimated variables, *h*_*i*_ and *f*_*i*_ (hit and false alarm rates), are shown as unshaded circular nodes. Further modelled (unobserved) parameters are also shown in the model – in particular, *c*_*i*_ and *d*_*i*_ which are estimates of bias and discriminability, and the top level of the hierarchy which formally incorporates group-wise mean (*μ*) and standard deviation (*σ*) estimates of both *c*_*i*_ and *d*_*i*_. To derive these estimates, signal and noise trials are denoted by S and N, respectively, to which individual (_*i*_) counts of hits (*H*_*i*_) and false alarms (*F*_*i*_) are derived and, subsequently, their rates (hit rate = *h*_*i*_ and false alarm rate = *f*_*i*_). In the graphical model, *ϕ* is used to calculate the cumulative distribution function of *h*_*i*_ and *f*_*i*_, whilst *λ* (at the top of the hierarchy) is the precision of the *c* and *d* parameters, from which *σ* estimates are obtained.

For bias estimates, the optimal criterion is centred at zero between two equal-variance Gaussian distributions representing signal and noise distributions. A negative bias value relative to zero indicates a preference towards more ‘yes’ responses (as it is nearer the noise distribution), whereas higher positive values reflect a preference for ‘no’ responses. The location of the response criterion (positioned along a unidimensional strength construct of perceptual accuracy) is derived from a participant’s estimate as to whether a stimulus constitutes a signal or a noise trial. For instance, a participant whose ‘yes rule’ is further from the noise distribution requires a stronger signal to permit detection – thus, the strength of the signal required is determined by the strength of the participant’s internal representation of that signal. In such cases, the available perceptual evidence needs to be substantially greater than the criterion rule to say ‘yes’ [14]. Discriminability is an estimate of the ability to differentiate between signal and noise trials and indicates how well a signal trial can be detected, with higher values indicating an increased capacity to distinguish between signal and noise trials. The previously developed implementation of hierarchical SDT [12], as shown in Additional file 1, was modified and implemented in WinBUGS [37]. Posterior distributions of bias and discriminability were estimated using MCMC sampling (10,000 samples), using hit and false alarm rates over uniform (reference) priors (i.e. where all responses are equally likely). The posterior means of bias and discriminability, taken as the joint emotional signal detection capacity of individuals in each group, is the focus of our analyses. Convergence of all chains was assessed using *R̂* (obtained by comparing two parallel chains in WinBUGS [38]) and using Geweke’s method [39], which compares the first 10 percent of each chain with the last 50 percent. All chains were found to be at convergence after 10000 iterations using *R̂* (all <1.1). However, the MCMC samples for the positive condition in the non-melancholic group did not converge using the criteria of Geweke [39] and, thus, a burn-in of 5000 samples (i.e. where the first 5000 samples are discarded, followed by a further 10,000 samples being drawn) for all positive chains across all groups was used.

The modelling approach allows for inspection of a range of posterior distribution statistics. The 95% credible interval (CI) estimates are reported for each of the individual distributions of bias and discriminability, which are compared according to a 95% highest posterior density (HPD) interval difference [40]. A 95% HPD group difference estimate (HPDd) that does not contain zero is considered “significantly different”. This latter approach is similar to that suggested by Lindley [41]. We also visualise the violin plots across modelled parameters in each group for each signal condition (using the MCMC distributions) (see Additional file 2). Violin plots marry the traditional box plot, representing the interquartile range, with smoothed distributional characteristics of the samples [42].

## Results

### Sample characteristics

Group differences on demographic variables were assessed using independent groups *t*-tests for continuous variables and chi-square statistics for categorical variables (α set at 0.05). Group characteristics including age, gender, depression severity, STAI scores, GAF, estimated IQ and medication status (SSRI and/or other medication) are presented in Table 1.

**Table 1:** Clinical and demographic characteristics of melancholic (Mel), non-melancholic (N-Mel) and control groups

**Test Variables**	**Mel**	**N-Mel**	**Control**	**Group Contrasts**
**Age**	41.7 (13.5)	42.4 (9.1)	38.6 (15.2)	§ *t* = -0.19, *p* = 0.85 ‡ *t* = 0.68, *p* = 0.85 † *t* = 0.96, *p* = 0.34
**% Female**	65%	65%	55%	§ *χ*^2^ = 0.00, p = 1.00 ‡ *χ*^2^ = 0.42, p = 0.52 † *χ*^2^ = 0.42, p = 0.52
**Years of education**	14.4 (2.7)	14.1 (2.7)	17.1 (3.7)	§ *t* = 0.43, *p =* 0.41 ‡ *t = -*2.66, *p* <0.01 † *t* = -3.00, *p* <0.01
**Estimated IQ**	108.1 (8.5)	109.2 (6.4)	114.3 (11.8)	§ *t* = -0.46, *p* = 0.65 ‡ *t* = -1.91, *p* = 0.06 † *t* = -1.70, *p* = 0.10
**QIDS-SR**^**16**^	16.6 (4.0)	16.6 (4.4)	0.9 (1.2)	§ *t* = 0.00, *p* = 1.00 ‡ *t* = 16.92, *p* <0.01 † *t* = 15.39, *p* <0.01
**STAI-State**	49.1 (15.3)	48.3 (9.7)	28.7 (8.1)	§ *t* = 0.19, *p* = 0.85 ‡ *t* = 5.28, *p* <0.01 † *t* = 7.00, *p* <0.01
**STAI-Trait**	59.1 (11.4)	62.9 (8.3)	34.2 (7.4)	§ *t* = -1.20, *p* = 0.24 ‡ *t* = 8.17, *p* <0.01 † *t* = 11.55, *p* <0.01
**CORE** ***(Non-interactiveness)***	4.4 (3.3)	0.9 (1.7)	-	§ *t* = 4.28, *p* <0.01
**CORE** ***(Retardation)***	4.9 (4.1)	1.6 (2.9)	-	§ *t* = 3.00, *p* <0.01
**CORE** ***(Agitation)***	1.5 (2.4)	0.2 (0.7)	-	§ *t* = 2.38, *p* < 0.05
**CORE Total**	10.8 (7.6)	2.7 (4.4)	-	§ *t* = 4.14, *p* <0.01
**GAF**	54.3 (15.6)	67.5 (9.7)	94.5 (2.2)	§ *t* = -3.23, *p* <0.01 ‡ *t = -*11.43, *p* <0.01 † *t* = -12.17, *p* <0.01
**SSRI%yes (n)**	30% (6)	40% (8)	-	§ *χ*^2^ = 9.69, p <0.01
**Other med%yes (n)**	80% (16)	35% (7)	-	§ *χ*^2^ = 27.22, p <0.01

Mean (SD) reported for all variables except for gender (% Female), SSRI (% yes and n) and Other med (% yes and n).

§ Melancholic vs. Non-melancholic.

‡ Melancholic vs. Control.

† Non-melancholic vs. Control.

There were no group differences for age, gender or estimated IQ. Both depressed groups had fewer years of education than the control group but did not differ from each other. Depression severity as measured by the QIDS, as well as STAI-State and STAI-Trait scores did not differ between melancholic and non-melancholic depression groups but, as anticipated, each clinical group differed significantly from the control group. Consistent with their classification, the melancholic group had significantly higher (CORE-rated) psychomotor disturbance scores compared to the non-melancholic group, as well as lower GAF scores (and also in comparison to controls). Lower rates of current selective serotonin reuptake inhibitor (SSRI) usage and higher rates of medication other than SSRI’s (e.g. tricyclics, serotonin noradrenaline reuptake inhibitors (SNRI’s)) were observed in the melancholic group compared to the non-melancholic group. Raw counts of hits, misses, false alarms and correct rejections across signal conditions in the AGN task are displayed in Table 2, along with signal detection sensitivity values, indexed by d-prime (*d*′).

**Table 2:** Frequencies of hits (H), misses (M), false alarms (FA) and correct rejections (CR) across signal valence conditions and group on the go/no-go task

**Positive**							
Group	H	M	FA	CR	Hit Rate	False Alarm Rate	*d*′
Melancholic depression	911	169	100	979	0.84	0.09	2.33
Non-Mel depression	979	101	141	939	0.91	0.13	2.47
Control	964	115	134	945	0.89	0.12	2.40
**Negative**							
Group	H	M	FA	CR	Hit Rate	False Alarm Rate	*d*′
Melancholic depression	1007	71	88	991	0.93	0.08	2.88
Non-Mel depression	1041	39	72	1007	0.96	0.06	3.31
Control	1008	70	83	997	0.93	0.07	2.95
**Neutral**							
Group	H	M	FA	CR	Hit Rate	False Alarm Rate	*d*′
Melancholic depression	809	271	213	864	0.75	0.20	1.52
Non-Mel depression	861	216	221	858	0.80	0.20	1.68
Control	908	171	177	903	0.84	0.16	1.99

*d*′ is presented as a function of hit and false alarm rates.

(NB: within conditions, not all participant’s scores summed to the total number of trials because pre-emptive responses were not recorded - however, *d*′ was calculated for the total number of trials).

Visual inspection of these frequency tables suggests higher hit rates in the non-melancholic and control groups compared with the melancholic group across positive and neutral conditions. The *d*′ statistics (indexing the separation of the signal and noise response distributions) also point to a reduction in signal detection sensitivity across all signal conditions in the melancholic group compared to the non-melancholic and control groups. To examine AGN task performance more formally we next report on the hierarchical Bayesian modelling.

### Group effects of mean bias and discriminability

**Figure 3:**
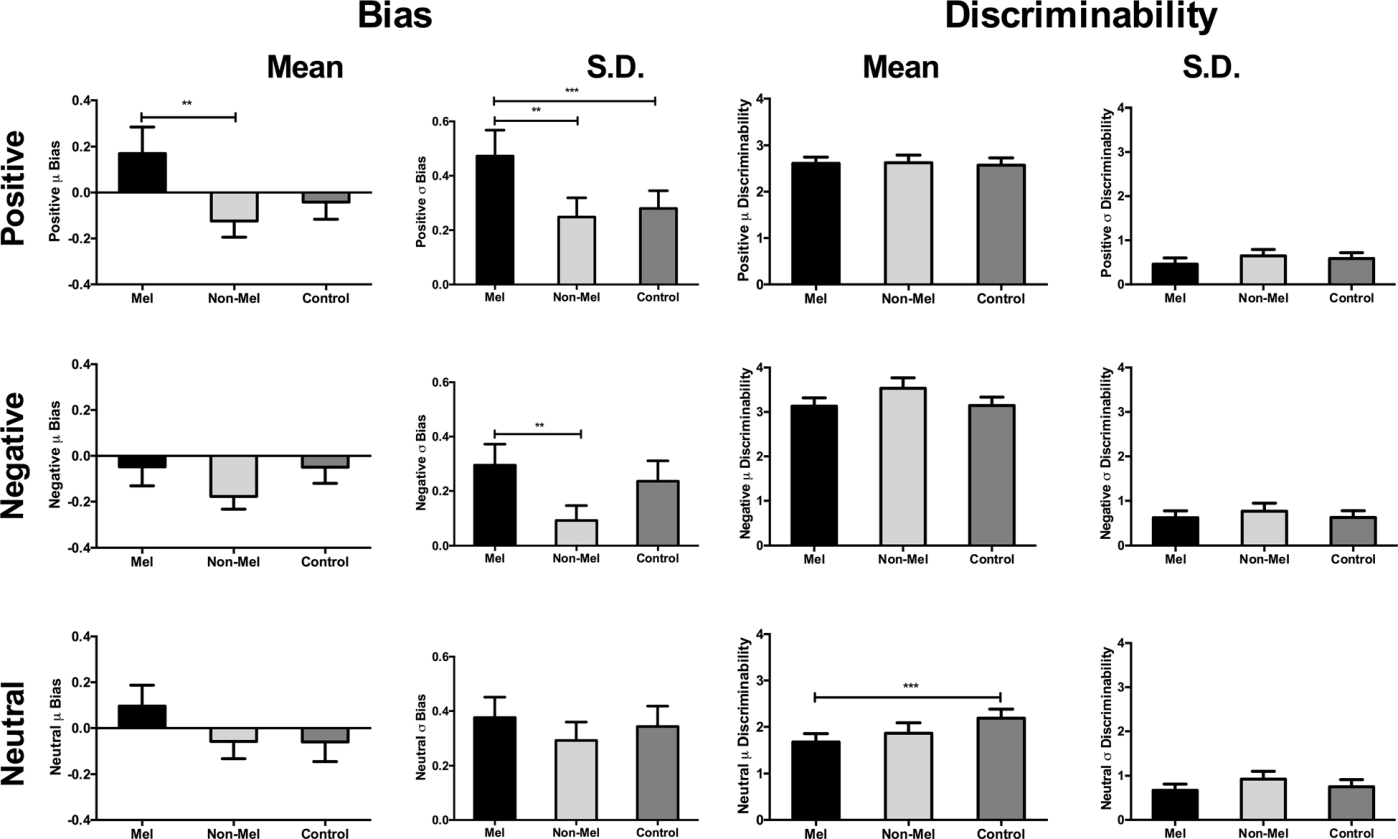
**Mean and standard deviation (SD) posterior estimates of bias and discriminability across groups.** Legend: Mel = melancholic, Non-Mel = non-melancholic. ** denotes difference between melancholic and non-melancholic groups. *** denotes difference between melancholic and control groups.

**Figure 4:**
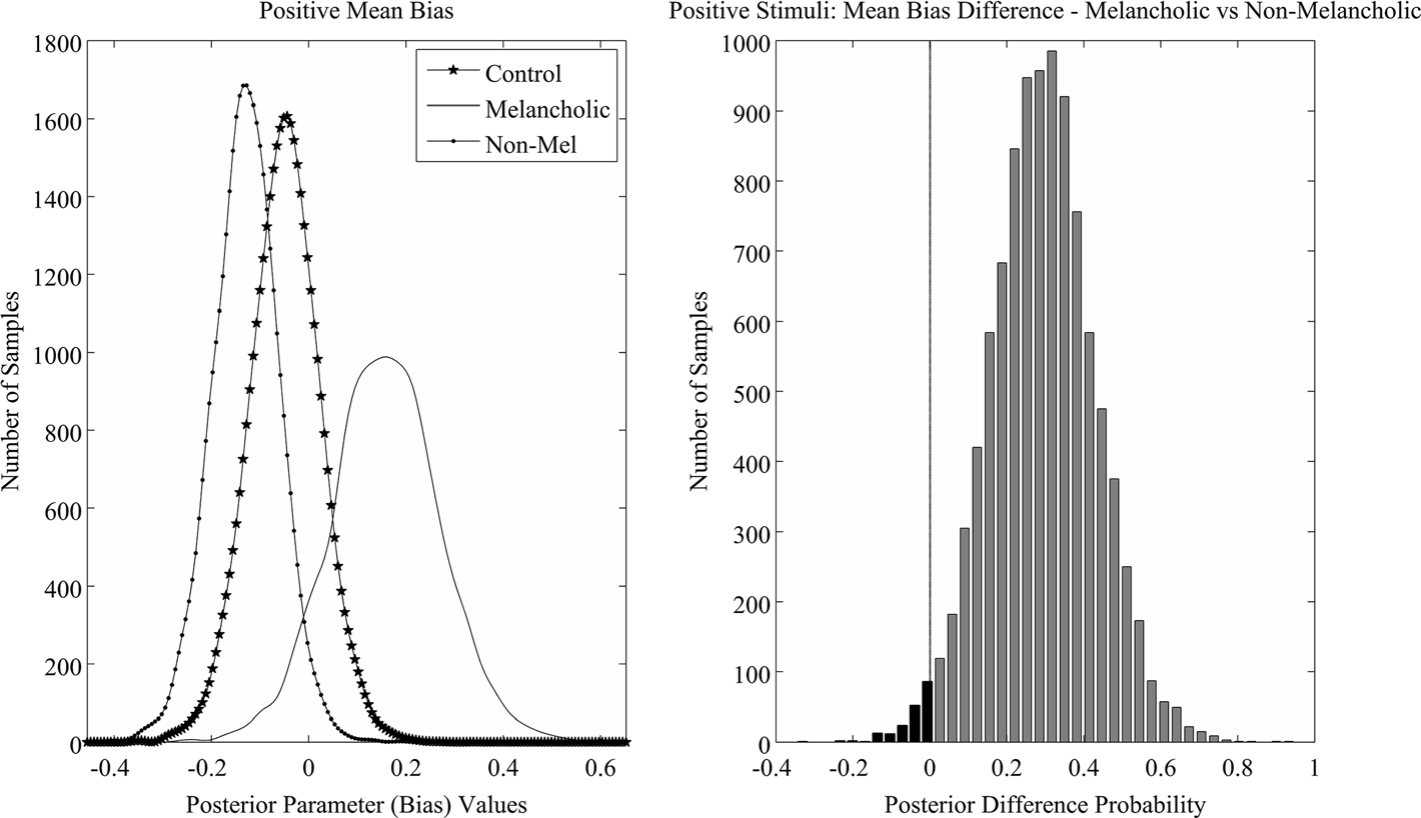
**Left: Posterior distributions of MCMC sampling for the mean bias to positive signal trials for each group.** Right: Posterior density of the estimated difference between melancholic and non-melancholic groups for the bias to positive signal trials – dashed line indicating the crossing of the difference distribution at zero.

**Figure 5:**
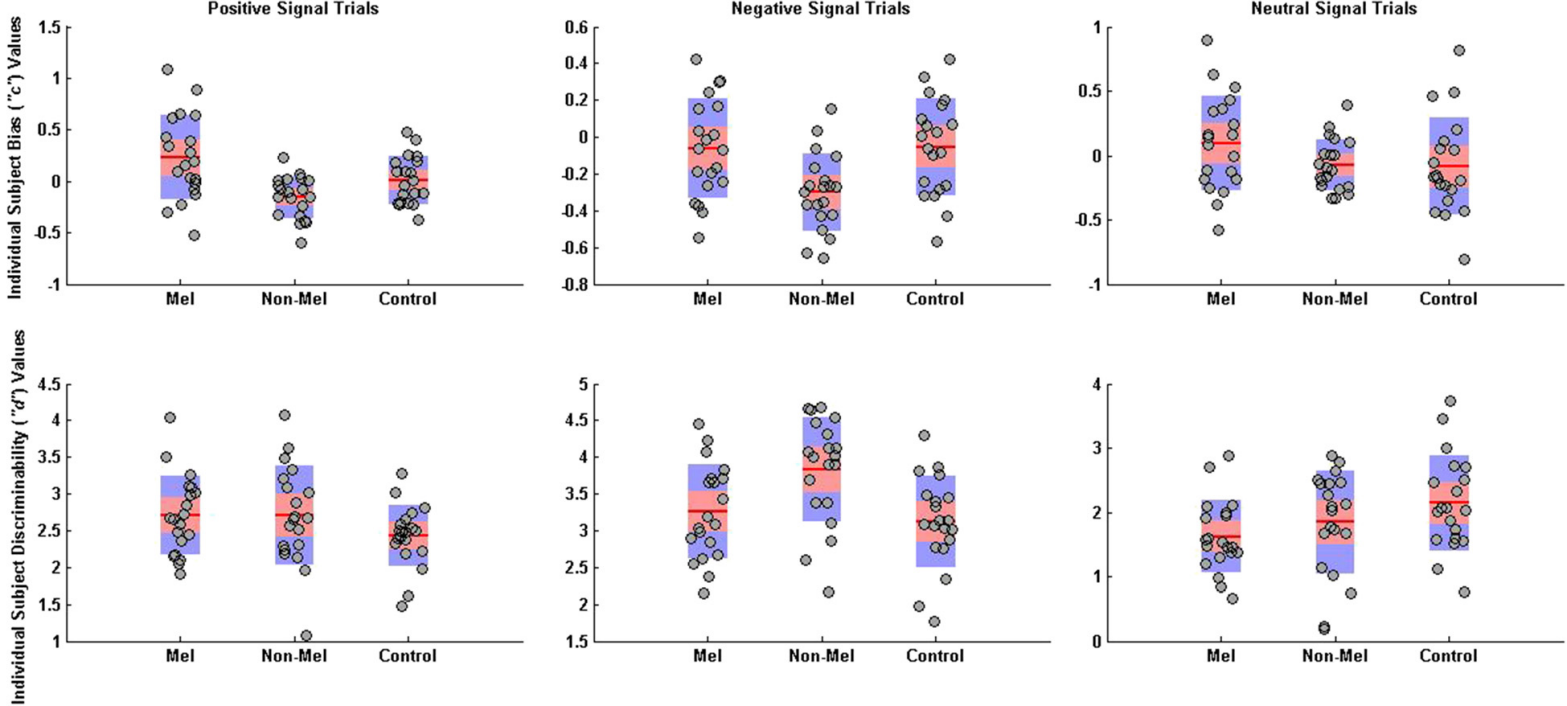
Individual parameter estimates for bias and discriminability to positive, negative and neutral signal conditions across groups.

Results from the hierarchical modelling showed a significant difference between melancholic and non-melancholic groups in terms of their mean bias to positive signal conditions (HPDd = 0.022 – 0.554). As shown in Figure 3, this difference was driven by the melancholic group favouring ‘no’ responses (positive mean bias values) and the non-melancholic group favouring ‘yes’ responses (negative mean bias values), but neither depressed group differed from the control group on this measure. This observed differential performance between depressed groups is further illustrated in Figure 4, with the left panel displaying the mean posterior estimates of bias for all groups and the right panel showing the posterior probability for the difference between melancholic and non-melancholic groups. The violin plots (Additional file 2) provide additional means for inspecting these differences. Individual parameter estimates for both bias and discriminability across the differing signal conditions are provided in Figure 5. Visualising the data in this manner suggests that the group results are a valid representation of the inter-subject variability and are not driven by outliers, clustering or multiple sub-groups.

In terms of mean discriminability, there was a significant difference between melancholic and control groups during neutral trials (HPDd = -1.020 – -0.042). This difference in posterior estimates of discriminability is consistent with impaired discrimination capacity for neutral signals in the presence of both positive and negative noise trials. The non-melancholic group appeared slightly less optimal than the control group in terms of discriminability to neutral signals, but this effect did not reach significance. Hierarchical analyses of the mean bias for negative and neutral signal conditions, and mean discriminability for positive and negative signal conditions did not reveal any differences across groups.

### Comparing standard deviation model estimates

Posterior estimates of the standard deviations for bias and discriminability across all stimulus conditions are also shown in Figure 3. Significantly increased standard deviation values were found in the melancholic group compared to non-melancholic and control groups on positive signal trials. A similar pattern was found for the negative signal condition, with larger standard deviation estimates found in the melancholic group compared to the non-melancholic group. No group effects were found for neutral bias or for any of the signal conditions for discriminability. The increased standard deviations on the emotional signal blocks in the melancholic group is indicative of greater variability of mean bias values across participants, thus reflecting a significantly broader distribution of bias values compared to those values observed in the other groups.

### Sensitivity and robustness analyses of model posteriors

We conducted a robustness check of the results using narrower priors on mean and gamma (precision) of bias and discriminability and, additionally, a sensitivity analysis by uncollapsing noise conditions. These additional analyses are presented in Additional file 3. Briefly, for the hierarchical model of positive and neutral signal conditions, differences in mean bias and discriminability were more robust to changes in prior distributions than were the standard deviation differences. The majority of effects were robust when the noise conditions were unpooled. The loss of some significant effects is consistent with the loss of power that arises when trials are split and not pooled. Nonetheless, these additional analyses do highlight that bias to positive signal trials, and discriminability to neutral signal trials, may be influenced by differing noise conditions.

## Discussion

The hierarchical Bayesian SDT model implemented in this paper revealed that signal detection processes in melancholic and non-melancholic depression are significantly influenced by stimulus type and individual subject variability. Our modelling approach allowed interrogation of the neuropsychological data at two levels: the mean results across individuals in specific groups, and the heterogeneity of the groups themselves, across the psychophysical constructs of bias and discriminability. In terms of mean differences, we observed that the melancholic participants overall were less sensitive to detecting emotional signals, while non-melancholic participants displayed more liberal responses to emotional signal blocks. This provides support for our predictions that the melancholic group would display difficulty in detecting signal trials from noise trials on emotional word blocks. Also, optimal responding was found to be reduced in the melancholic group compared to the control group, as evidenced by decreased mean discriminability on neutral signal blocks. In terms of subject heterogeneity, we found that there was greater inter-subject variability of the bias estimates for the emotional signal conditions in the melancholic group, which indicates divergent bias estimates across individuals. Visualising the range of individual participant responses (Figure 5) argues against this effect being driven by outliers. Further, changes to the precision of prior distributions had little impact on the observed mean difference findings, suggesting these findings are highly robust. Despite this, however, when differing noise conditions were examined there was a moderate effect, with some previously significant differences for specific signal conditions no longer remaining significant. The observed impact of differing noise conditions thus warrants further consideration in future psychophysical studies using differing emotional and non-emotional stimuli. The main findings allow for specific neurocognitive models to be advanced with regard to depression and its sub-typing, namely the potential to gain insight into underlying psychophysical mechanisms across individuals and whether the depression is melancholic or non-melancholic in type, and highlight several important issues in the analysis and interpretation of neurocognitive data.

Our findings align with the commonly held notion (e.g. [3]) that those with melancholic depression exhibit cognitive deficits that are observable during task performance. However, we add the observation that those with non-melancholic depression may also be impaired in their ability to perform ‘optimally’ on cognitive tasks such as the AGN. The observed trend of less optimal responding in non-melancholic depression did not, however, reach significance but may benefit from a focus in future studies. While research using the AGN task in depression [43] claims it as a measure of ‘inhibitory control’, the analytic methods previously employed often prevent interpretation beyond a continuum of impairment (e.g. number of errors on a task). The current study is the first, to our knowledge, to utilise an affective go/no-go task in sub-types of depression. In doing so – and through analysis of the data using hierarchical Bayesian SDT – the findings offer an increased understanding of the sensitivity and discriminability capacity of individuals with differing types of depression, and highlight the importance of examining for apparent dysfunction with more refined models. Recently, Schulz and colleagues [44] examined the convergent validity of emotional and non-emotional go/no-go tasks and concluded that, together, they offer “moderate capacity” for probing the neuropsychological construct of behavioural inhibition. Those authors also emphasized the need for testing emotional and non-emotional signal detection mechanisms in affective disorders to clarify underlying cognitive-emotional contributions. The diverging sensitivities across emotional and non-emotional conditions in the depressed groups in the current study, along with a lack of discrimination to neutral signals in melancholia, suggests that a range of cognitive mechanisms may be involved in responding to differing stimuli.

Across neuropsychological studies of depression it is evident that no single cognitive deficit model can be applied to specific groups, due to the inherent heterogeneity of the depressive domain. However, in light of the current findings of discrepancies in bias between signal conditions, it might be possible that set-shifting impairments – as previously reported in depression [43], and more specifically in melancholic depression [45] – play an important role. While not explicitly assessed in the current study, it is conceivable that neuropsychological constructs such as disrupted attention set-shifting and perseveration underlie the observed effects. The melancholic sub-type has been shown to be differentiated from non-melancholic depression on the basis of response selection performance [46], where performance on compatible and incompatible trial types (e.g. stimulus–response compatibility tests) reflect cognitive strengths and weaknesses. As a rule these studies have not specified psychophysical functions of stimulus sensitivity and discrimination capacity, and have tended to report broader metrics of performance such as numbers of hits and misses across subjects. From a cognitive standpoint there is likely to be significant benefit in modelling performance-related psychophysical mechanisms (i.e. through SDT and similar analyses) in depressive disorders and then next establishing whether this provides insight towards any neurocognitive disease mechanisms. We argue that the cognitive deficits observed in different types of depression can be conceptualised in such a way so as to explain impairments in emotion-bound optimal decision-making.

Prior research on sensory processing offers further insight into the findings of decreased sensitivity to emotional signals and poorer discrimination to neutral signals in melancholia. Knill and Pouget [9] suggest that perception of one’s environment is influenced by the likelihood of the presence or absence of relevant stimuli given an individual’s past experience (i.e. perceptual priors) with that stimuli. These factors contribute to the *relative* uncertainty over one’s environment, and allow inference regarding the causes of percepts. We propose that the low sensitivity to emotional signals and lack of discriminability observed across melancholic participants may be a result of inefficient sensory integration – possibly resulting from constructs such as inefficient cognitive control mechanisms (see [47]). This in turn may be a function of ‘inflexible’ priors (e.g. negative cognitive biases) and unsuccessful updating (e.g. such as perseveration due to a failure of emotional inhibition [48]). The observation that the non-melancholic participants responded more frequently to noise trials on emotional signal blocks also suggests that they too are less sensitive to fluctuations in the emotional environment. Such erroneous judgements could be due to emotional processing biases in depression, a factor that has been acknowledged in accounting for decision-making impairments [5]. Research into the probabilistic nature of decision-making [21,49,50] suggests diverse mechanisms underlying optimal judgement. Neurobiologically, probabilistic learning paradigms have been used to examine human cognition [51], with the findings pointing to distinct roles of serotonin and noradrenaline in learning and inhibition. Both neurochemicals have long been implicated in depressive disorders [52] and may be of relevance to understanding the differences observed between and within depressive sub-types.

In addition to the behavioural changes within individuals, research using Bayesian inference has also highlighted the importance of perceptual variation between individuals. Recent theoretical work on perceptual uncertainty advocates the utility of modelling trial-by-trial updating across individuals in a Bayesian framework [53], which is argued to be of significant benefit in conceptualising the current findings from the signal detection task. The hierarchical modelling using MCMC in the current study yielded estimates of the standard deviation of both bias and discriminability performance (i.e. the extent of the differences between measured individuals). The increased standard deviations in the melancholic group on emotional bias suggests differential individual performance profiles when compared to non-melancholic and control groups. This could be due to a range of non-cognitive factors in an otherwise homogeneous group or may alternatively reflect divergent cognitive strengths and weaknesses, which we now consider.

Several lines of research have indicated that melancholic depression is associated with diurnal variation of mood [54], with such variation thought to impact on neuropsychological performance across the day [55]. Clinical depression with diurnal variation has also been found to result in differential performance on accessibility and recall of positive and negative (self-related) experiences [56], with positive memories more likely to be retrieved when depression is less severe in the afternoon/evening. Varying biological influences such as cortisol hypersecretion – shown to be specific to melancholia [57] – may play a pivotal role in modulating cognitive function in depression as previously suggested [58]. Such factors are important considerations with respect to the current findings given study participants were not all tested at the same time of day. Furthermore, inconsistent medication regimens across individuals within and between groups may contribute to individual differences in emotional processing biases [59], thus possibly dampening the association between the depressed state and cognitive impairments. If such factors were found to be unrelated – and individual differences were indeed evident upon replication – it is conceivable that melancholia (due to the observed variation) may be able to be portrayed as comprising several distinct sub-types as suggested by Parker and Hadzi-Pavlovic [1] (e.g. functional and structural melancholia). Factors such as family history, age-of-onset, presence/absence of neuropathological changes and cardiovascular disease would need to be clarified for any such sub-typing model to be advanced within the current context. Given the increased age of our sample, neuropathological changes in some individuals cannot be excluded. Despite these possibilities, the utility of the current findings lie first and foremost in their ability to inform psychophysical models of depression, with several caveats.

As indicated above, there were several study limitations. Firstly, the analysis did not interrogate trial-to-trial variability. Thus, the supposed dynamics of sensory integration (stimulus–response updating), as previously put forward [53], could not be quantified in this sample. In addition, the signal detection task used did not allow for further analysis of aspects of emotional decision-making (i.e. specific decision-making rules) beyond bias and discriminability functions. Such specific limitations, if overcome in future studies, would provide a more refined model of decision-making and clinical conditions themselves. It is therefore proposed that future work should attempt to examine the utility of dynamic models of decision-making in light of changing emotional environments, along with key clinical variables, to further establish the mechanisms by which perception and action interact in depression.

## Conclusions

There has been an upsurge of interest in framing cognitive function in psychiatric conditions in probabilistic terms [5,60], precipitated by research in cognitive and computational sciences that, in health, humans respond optimally in their environments. The signal detection approach used in the current study offers insight into the optimal response parameters of those with depression, and extends previous suggestions of ‘emotional response biases’ in depression through psychophysical modelling. The hierarchical models in the current paper allowed estimation of the most probable response distributions, and is an advance on previous (e.g. frequentist) attempts that aim to elucidate neuropsychological dysfunction in depressed groups through group averaging approaches. Future studies should attempt to clarify how different cognitive processes operate across different individuals. Such work should also aim to provide a more detailed characterization of perceptual (probabilistic) sensory updating across changing environments in depression, whilst recognising the fundamental role pre-existing cognitive biases play in response to environmental demands.

## Abbreviations

AGN: Affective Go/No-go Task; CANTAB: Cambridge Neuropsychological Test Automated Battery; CORE: The CORE Assessment of Psychomotor Change; GAF: Global Assessment of Functioning; HPD: Highest Posterior Density; HPDd: Highest Posterior Density Difference; IED: Intra/Extra-Dimensional Shift; ISI: Inter-Stimulus Interval; MCMC: Markov chain Monte Carlo; MINI: Mini-International Neuropsychiatric Interview; QIDS-SR16: 16-item Quick Inventory of Depressive Symptomatology; RVP: Rapid Visual Information Processing; SDT: Signal Detection Theory; SOC: Stockings of Cambridge; SNRI: Serotonin-Noradrenaline Reuptake Inhibitor; SSRI: Selective Serotonin Reuptake Inhibitor; STAI: State-Trait Anxiety Inventory; WAIS-III: Wechsler Adult Intelligence Scale – Third Edition; WinBUGS: Windows Bayesian inference Using Gibbs Sampling; WTAR: Wechsler Test of Adult Reading.

## Competing interests

There are no competing interests for any of the authors.

## Authors’ contributions

MH undertook neuropsychological testing of participants, undertook the analyses and wrote the final version of the manuscript. GP held primary responsibility for overseeing clinical diagnoses and contributed to writing the manuscript and was the chief investigator on the study from which the data arose. MB contributed to the statistical modelling and interpretation of the results and assisted in the drafting of the manuscript. All authors read and approved the final manuscript.

## Pre-publication history

The pre-publication history for this paper can be accessed here:

http://www.biomedcentral.com/1471-244X/14/122/prepub

## Supplementary Material

Additional file 1 Violin plots (overlaid with box-plots) of posterior distributions of the mean and standard deviation of bias and discriminability across signal conditions and groups. Click here for file

Additional file 2 Hierarchical Signal Detection Theory. Click here for file

Additional file 3 Sensitivity and robustness analyses of model posteriors. Click here for file
