# Supplementary material for: Bias and discriminability during emotional signal detection in melancholic depression
Source: BMC Psychiatry. 2014 Apr 27;14:122. doi: 10.1186/1471-244X-14-122 (PMC4022535; doi:10.1186/1471-244X-14-122)
Supplement: Additional file 1 — Violin plots (overlaid with box-plots) of posterior distributions of the mean and standard deviation of bias and discriminability across signal conditions and groups. [file 1471-244X-14-122-S1.pdf]

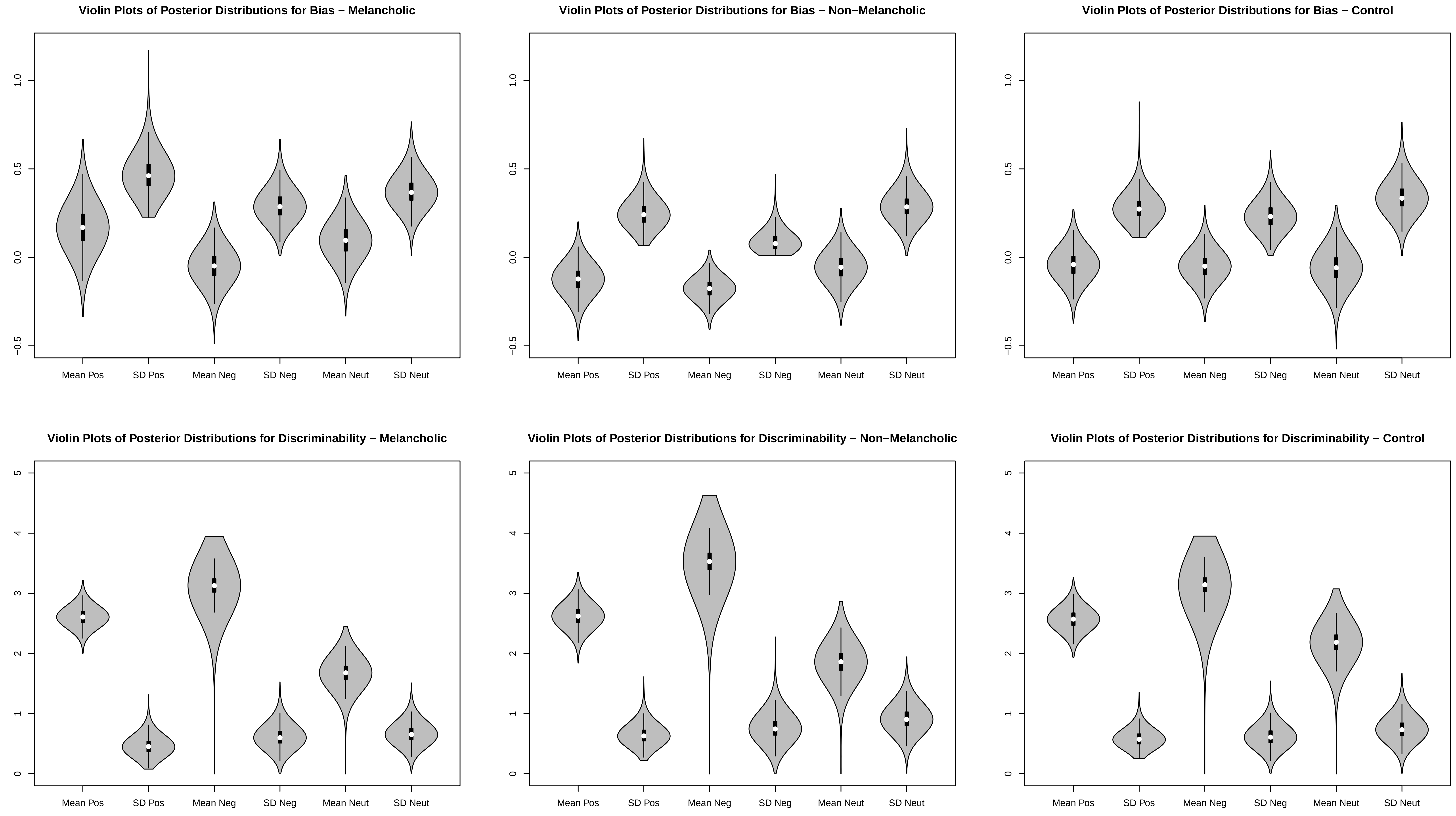
Violin Plots of Posterior Distributions for Bias − Melancholic
Violin Plots of Posterior Distributions for Bias − Non−Melancholic
Violin Plots of Posterior Distributions for Bias − Control
Violin Plots of Posterior Distributions for Discriminability − Melancholic
Violin Plots of Posterior Distributions for Discriminability − Non−Melancholic
Violin Plots of Posterior Distributions for Discriminability − Control
−0.5
0.0
0.5
1.0
0
1
2
3
4
5
Mean Pos
SD Pos
Mean Neg
SD Neg
Mean Neut
SD Neut
