# Supplementary material for: Bias and discriminability during emotional signal detection in melancholic depression
Source: BMC Psychiatry. 2014 Apr 27;14:122. doi: 10.1186/1471-244X-14-122 (PMC4022535; doi:10.1186/1471-244X-14-122)
Supplement: Additional file 2 — Hierarchical Signal Detection Theory. [file 1471-244X-14-122-S2.doc]

**Additional File 2**

# Hierarchical Signal Detection Theory

model{

# Relating observed counts to underlying Hit and False Alarm rates

for (i in 1:n) {

HR[i] ~ dbin(h[i],S[i])

FA[i] ~ dbin(f[i],N[i])

S[i] <- HR[i]+MI[i]

N[i] <- FA[i]+CR[i]

}

# Reparameterization Using SDT

for (i in 1:n) {

h[i] <- phi(d[i]/2-c[i])

f[i] <- phi(-d[i]/2-c[i])

}

# Group Distributions

for (i in 1:n) {

c[i] ~ dnorm(muc,lambdac)

d[i] ~ dnorm(mud,lambdad)

}

# Priors

muc ~ dnorm(0,.001)

mud ~ dnorm(0,.001)

lambdac ~ dgamma(.001,.001)

lambdad ~ dgamma(.001,.001)

sigmac <- 1/sqrt(lambdac)

sigmad <- 1/sqrt(lambdad)

}
