# Supplementary material for: Bias and discriminability during emotional signal detection in melancholic depression
Source: BMC Psychiatry. 2014 Apr 27;14:122. doi: 10.1186/1471-244X-14-122 (PMC4022535; doi:10.1186/1471-244X-14-122)
Supplement: Additional file 3 — Sensitivity and robustness analyses of model posteriors. [file 1471-244X-14-122-S3.doc]

**Additional File 3**

**Sensitivity and robustness analyses of model posteriors**

Two separate sensitivity analyses were conducted to determine whether our significant group differences were upheld under differing prior assumptions. The first analysis was a respecification of the hierarchical models using different priors, namely narrower (more precise) priors for muc and mud (mean of zero and precision of 0.01) and also for lambdac and lambdad (set at 0.01,0.01). Whilst we had no clear prior knowledge to inform our choice of these parameter values – hence the original use of uniformative priors – these are important checks of robustness. For the second sensitivity analysis we uncollapsed the noise conditions for those results that showed a between group effect.

***Impact of modifying precision of prior distributions on posterior differences***

The main effects of positive mean bias between melancholic and non-melancholic groups (HPDd = 0.024 – 0.559) were robust to the precision of the priors as were the neutral mean discriminability between melancholic and control groups (HPDd = -1.034 – -0.041). As the precision of the priors increased, the group differences for the standard deviation estimates crossed below statistical significance. This indicates that for the hierarchical model of positive and neutral signal conditions, differences on mean bias and discriminability are more robust to changes in the precision of the prior distributions than estimates of their variability.

***Effect of differing noise conditions***

When the uncollapsed noise conditions for the positive mean bias condition were examined across melancholic and non-melancholic groups, the positive/negative (signal/noise) condition remained significant (HPDd = 0.043 – 0.624), but there was no such significant effect for positive/neutral. For mean discriminability of neutral signal trials, the difference for the neutral/positive condition (HPDd = -1.109 – -0.098) remained significant, but not the group effect for the neutral/negative condition. Model differences for standard deviation of bias estimates between melancholic and non-melancholic groups were upheld across uncollapsed conditions, for both positive/negative (HPDd = 0.022 – 0.624) and positive/neutral (HPDd = 0.064 – 0.640). However, for negative signal trials the melancholic vs. non-melancholic group difference for the standard deviation of diminished when noise trials were not collapsed. When comparing melancholic and control groups the positive/negative contrast retained significance (HPDd = 0.028 – 0.610), but the positive/neutral condition did not show group differences. These effects highlight that bias to positive, and discriminability to neutral, signal trials may be influenced by differing noise conditions.
